# Correction: China Tuberculosis Policy at Crucial Crossroads: Comparing the Practice of Different Hospital and Tuberculosis Control Collaboration Models Using Survey Data

**DOI:** 10.1371/journal.pone.0099150

**Published:** 2014-05-30

**Authors:** The PLOS ONE Staff

There is information missing from Table 5. Please see the complete, corrected Table 5 here.

**Table 5:** Hospitalization of TB patients in the three models.

	The Dispensary Model			The Specialist Model			The Integrated Model		
	ZD	GP	Sub-total	SL	SDC	Sub-total	SC	GN	Sub-total
Patient survey	51	49	100	44	46	90	50	53	103
Total hospitalization,N (% of total patients) †	33(64.7)	6(12.2)	39(39.0)	40(90.9)	35(76.1)	75(83.3)	8(16.0)	7(13.2)	15(14.6)
Inpatient days (M) ‡	19	12	15	35	30	35	15	20	20
Inpatient expenditure(M, RMB)	5,030	3,815	5030	9,028	8,554	8,900	4,010	6,000	4,120
Hospitalization between TB diagnosis and DOTS treatment ,N (% of total hospitalization) §	30(58.8)	3(6.1)	33(33.0)	37(84.1)	23(50.0)	60(66.7)	3(6.0)	2(3.8)	5(4.9)
Inpatient days (M)	19	17	18	35	30	33	15	18	15
Inpatient expenditure(M, RMB)	4,905	8,597	4,905	9,150	6,450	7,960	2,050	4,277	2,554

1 USD = 6.8 RMB, M =  Mean.

† ZD was significantly higher than GP (χ2 = 29.910, P<0.001), SC (χ2 = 24.836, P<0.001) and GN (χ2 = 29.122, P<0.001). SL was significantly higher than ZD (χ2 = 9.114, P = 0.003), GP (χ2 = 57.389, P<0.001), SC (χ2 = 52.556, P<0.001) and GN (χ2 = 58.115, P<0.001). SDC was significantly higher than GP (χ2 = 39.420, P<0.001), SC (χ2 = 34.980, P<0.001) and GN (χ2 = 39.862, P<0.001).

‡ Significant difference was found among six sites (F = 4.034, P = 0.004). SL was significantly longer than GN (P = 0.028).

§ ZD was significantly higher than GP (χ2 = 31.392, P<0.001), SC (χ2 = 32.025, P<0.001) and GN (χ2 = 36.975, P<0.001). SL was significantly higher than ZD (χ2 = 7.255, P = 0.007), GP (χ2 = 57.495, P<0.001), SDC (χ2 = 11.761, P = 0.001), SC (χ2 = 58.385, P<0.001) and GN (χ2 = 64.510, P<0.001). SDC was significantly higher than GP (χ2 = 22.979, P<0.001), SC (χ2 = 23.487, P<0.001) and GN (χ2 = 27.879, P<0.001).
